# Supplementary material for: Enhancement of the Immunostimulatory Effect of Phosphodiester CpG Oligodeoxynucleotides by an Antiparallel Guanine-Quadruplex Structural Scaffold
Source: Biomolecules. 2021 Nov 1;11(11):1617. doi: 10.3390/biom11111617 (PMC8615816; doi:10.3390/biom11111617)
Supplement: Supplementary file 1 [file biomolecules-11-01617-s001.zip › biomolecules-11-01617_supp v1.pdf]

Enhancement of the Immunostimulatory Effect of Phosphodiester CpG Oligodeoxynucleotides by an Antiparallel Guanine-Quadruplex Structural Scaffold

Fika Ayu Safitri[1,2,] Anh Thi Tram Tu[2,3], Kazuaki Hoshi[2], Miwako Shobo[2], Dandan Zhao[2], Arief Budi Witarto[4], Sony Heru Sumarsono[5], Ernawati Arifin Giri-Rachman[5], Kaori Tsukakoshi[6], Kazunori Ikebukuro[6], and Tomohiko Yamazaki[2,3*]

[1] Doctoral Program in Biology, School of Life Sciences and Technology, Institut Teknologi Bandung (ITB), Bandung 40132, West Java, Indonesia; fikaayusafitri1904@gmail.com (F.A.S.)

[2] Research Center for Functional Materials (RCFM), National Institute for Materials Sci-ence (NIMS), 1-2-1, Sengen, Tsukuba, Ibaraki 305-0047, Japan; TU.ThiTramAnh@nims.go.jp (A.T.T.T.); k.hoshi.oe@juntendo.ac.jp (K.H.); SHOBO.Miwako@nims.go.jp (M.S.); ZHAO.Dandan@nims.go.jp (D.Z.)

[3] Division of Life Science, Graduate School of Life Science, Hokkaido University, Kita 10, Nishi 8, Kita-ku, Sap-poro 060-0808, Japan

[4] Department of Molecular Biology and Biochemistry, Faculty of Medicine, Indonesia Defense University, Ka-wasan IPSC Sentul, Sukahati, Kec. Citeureup, Bogor, Jawa Barat 16810, Indonesia; arief.witarto@idu.ac.id (A.B.W.)

[5] Physiology, Developmental Biology and Biomedical Sciences Research Group, School of Life Sciences and Technology, ITB, Bandung 40132, West Java, Indonesia; sonyheru@sith.itb.ac.id (S.H.S.); erna@sith.itb.ac.id (E.A.G.-R.)

[6] Department of Biotechnology and Life Science, Tokyo University of Agriculture and Technology, 2-24-16, Naka-cho, Koganei 184-8588, Japan; k-tsuka@cc.tuat.ac.jp (K.T.); ikebu@cc.tuat.ac.jp (K.I.)

*Correspondence: YAMAZAKI.Tomohiko@nims.go.jp (T.Y.); Tel.: +81-29-859-2345.

Fax: +81-29-859-24491

Supplementary Information

Figure and Table

Table S1. G4 CpG ODNs having three flanking sequences on the 5' and 3 'termini of CpG motifs

**Name**	**Sequence (5′-3′)**						
		**1st Loop**		**2nd loop**		**3rd loop**	
Gcac3aca.0.0	GTGACGTAGG	*ACA*GTCGTTTTGTCGTT*CAC*	GG	TGT	GG	TT	GGGGCGTCAC
Gcac3cac.0.0	GTGACGTAGG	*CAC*GTCGTTTTGTCGTT*CAC*	GG	TGT	GG	TT	GGGGCGTCAC

Note: CpG motifs are underlined. All ODNs have a phosphodiester backbone.

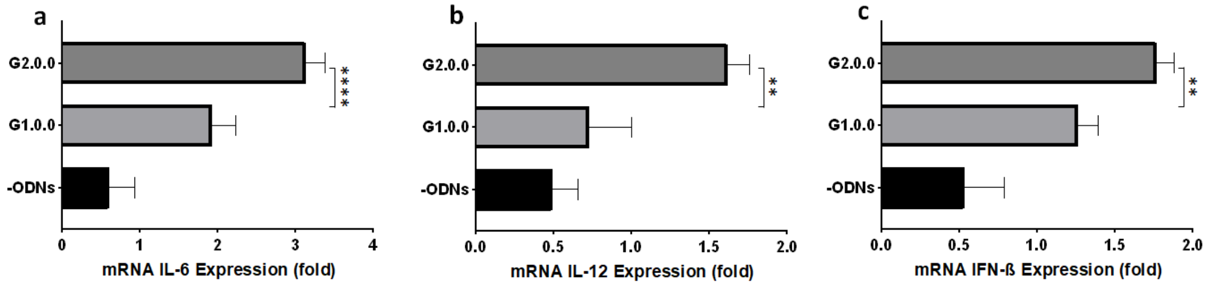

Figure S1. Effect of the number of CpG motifs on cytokine induction. Relative expression of (**a**) IL-6, (**b**) IL-12, and (**c**) IFN-β in mouse macrophage-like RAW 264 cells stimulated with G1.0.0, G2.0.0, and -ODNs. Data are presented as the mean ± SD (n=5). **** $p<0.0001$, *** $p<0.001$, ** $p<0.01$, * $p<0.05$, ns: not significantly different, (one-way ANOVA, Tukey's multiple comparisons test for comparison with other groups).

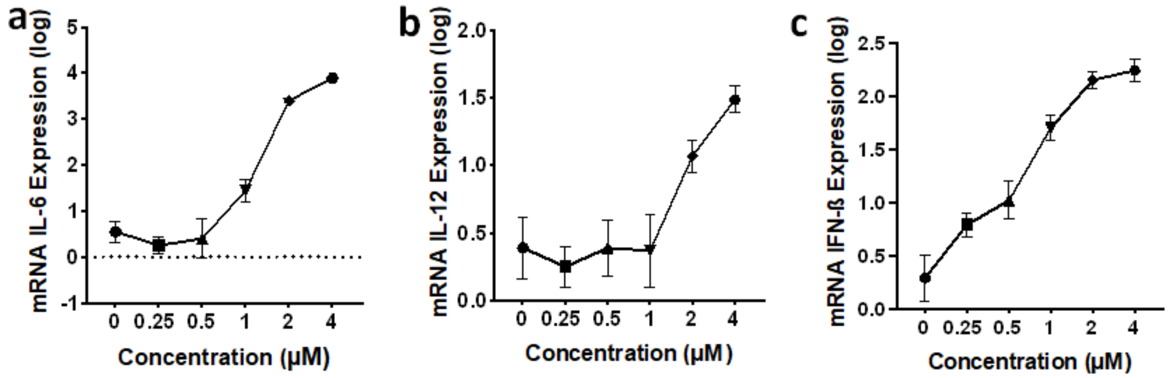

Figure S2. Relative expression levels of (**a**) IL-6, (**b**) IL-12, and (**c**) IFN-ß in mouse macrophage-like RAW 264 cells stimulated with different concentrations of G2.0.0 (0 µM, 0.25 µM, 0.5 µM, 1 µM, 2 µM, and 4 µM). Data are presented as the mean ± SD (n=5).

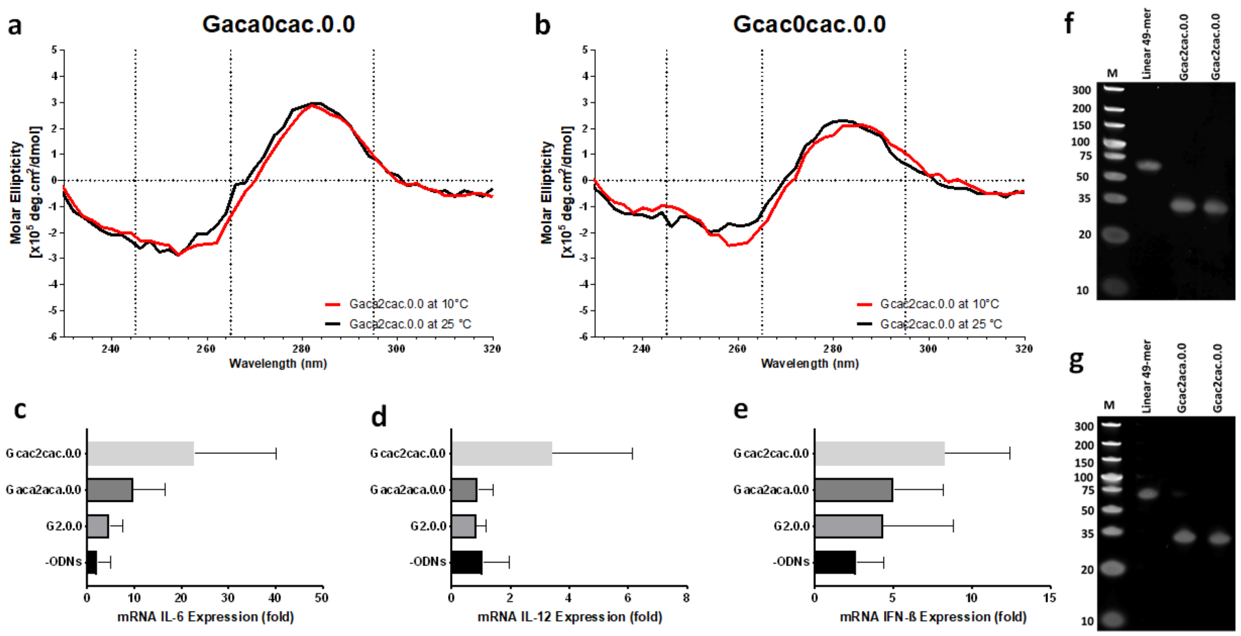

Figure S3. Effect of three connectors between CpG number and G-tetrad at first loop on antiparallel G-quadruplex formation and cytokine induction. Characterization of (**a**) Gaca2cac.0.0 and (**b**) Gcac2cac.0.0 was performed using circular dichroism (CD) analysis at 10 °C and 25 °C. Relative expression levels of (**c**) IL-6, (**d**) IL-12, and (**e**) IFN-ß in mouse macrophage-like RAW 264 cells stimulated by G2.0.0, Gcac2aca.0.0, Gcac2cac.0.0, and -ODNs. Mobility of CpG oligonucleotides reconstructed in 1× D-PBS buffer (4 mM K^+, 150 mM Na^+) and Tris-HCl in (f) non-denaturing 12% polyacrylamide gel in 0.5× Tris-Borate-EDTA (TBE) buffer containing 4 mM K^+; (g) denaturing 12% polyacrylamide gel in 0.5× TBE buffer. Data are presented as the mean ± SD (n=5). **** $p<0.0001$, *** $p<0.001$, ** $p<0.01$, * $p<0.05$, ns: not significantly different, (one-way ANOVA, Tukey's multiple comparisons test for comparison with other groups).

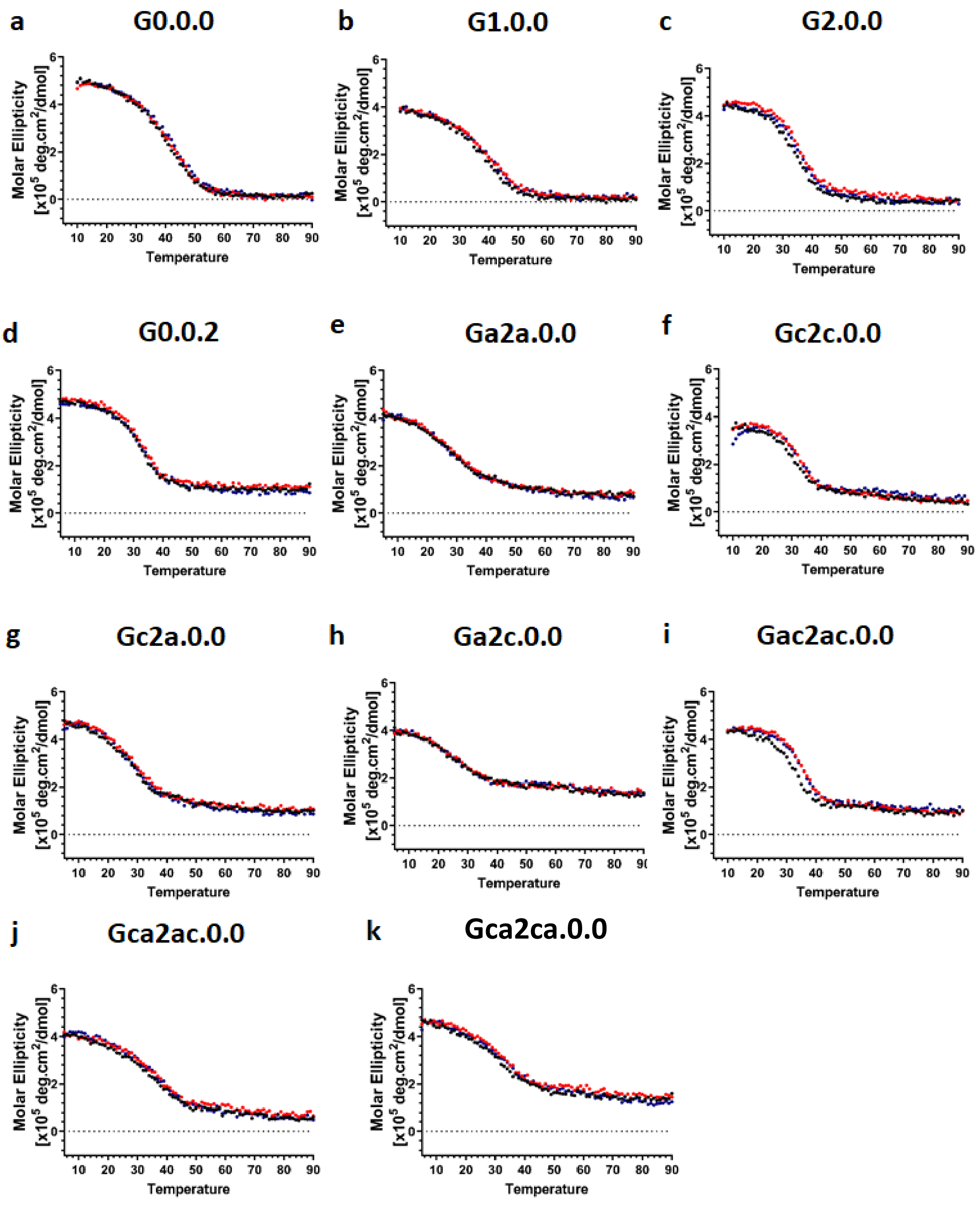

Figure S4. Melting temperature (Tm Value) analysis using CD-melting profile of 2 μM of (**a**) G0.0.0, (**b**) G1.0.0, (**c**) G2.0.0, (**d**) G0.0.2, (**e**) Ga2a.0.0, (**f**) Gc2c.0.0, (**g**) Ga2c.0.0, (**h**) Gc2a.0.0, (**i**) Gac2ac.0.0, (**j**) Gca2ac.0.0, and (**k**) Gca2ca.0.0 in 1× D-PBS buffer (pH 7.4) in the presence of 4 mM K^+ and 150 mM Na^+ at 295 nm. Data recorded with temperature changing 1 °C/min from 5 °C/10 °C to 90 °C known as 1st heating (Black colour); from 90 °C to 5 °C /10 °C known as 1st cooling (red colour); and blue colour is 2nd heating.
